# Molecular mechanism responsible for sex differences in electrical activity of mouse pancreatic **β** cells

**DOI:** 10.1172/jci.insight.171609

**Published:** 2024-02-15

**Authors:** Noelia Jacobo-Piqueras, Tamara Theiner, Stefanie M. Geisler, Petronel Tuluc

**Affiliations:** Department of Pharmacology and Toxicology, Institute for Pharmacy, University of Innsbruck, Innsbruck, Austria.

**Keywords:** Cell biology, Endocrinology, Diabetes, Insulin, Ion channels

## Abstract

In humans, type 2 diabetes mellitus shows a higher prevalence in men compared with women, a phenotype that has been attributed to a lower peripheral insulin sensitivity in men. Whether sex-specific differences in pancreatic β cell function also contribute is largely unknown. Here, we characterized the electrophysiological properties of β cells in intact male and female mouse islets. Elevation of glucose concentration above 5 mM triggered an electrical activity with a similar glucose dependence in β cells of both sexes. However, female β cells had a more depolarized membrane potential and increased firing frequency compared with males. The higher membrane depolarization in female β cells was caused by approximately 50% smaller K_v_2.1 K^+^ currents compared with males but otherwise unchanged K_ATP_, large-conductance and small-conductance Ca^2+^-activated K^+^ channels, and background TASK1/TALK1 K^+^ current densities. In female β cells, the higher depolarization caused a membrane potential–dependent inactivation of the voltage-gated Ca^2+^ channels (Ca_V_), resulting in reduced Ca^2+^ entry. Nevertheless, this reduced Ca^2+^ influx was offset by a higher action potential firing frequency. Because exocytosis of insulin granules does not show a sex-specific difference, we conclude that the higher electrical activity promotes insulin release in females, improving glucose tolerance.

## Abstract 2

Pancreatic β cells from female mice respond to glucose with a stronger membrane depolarization and a higher electrical activity than those from males.

## Graphical abstract

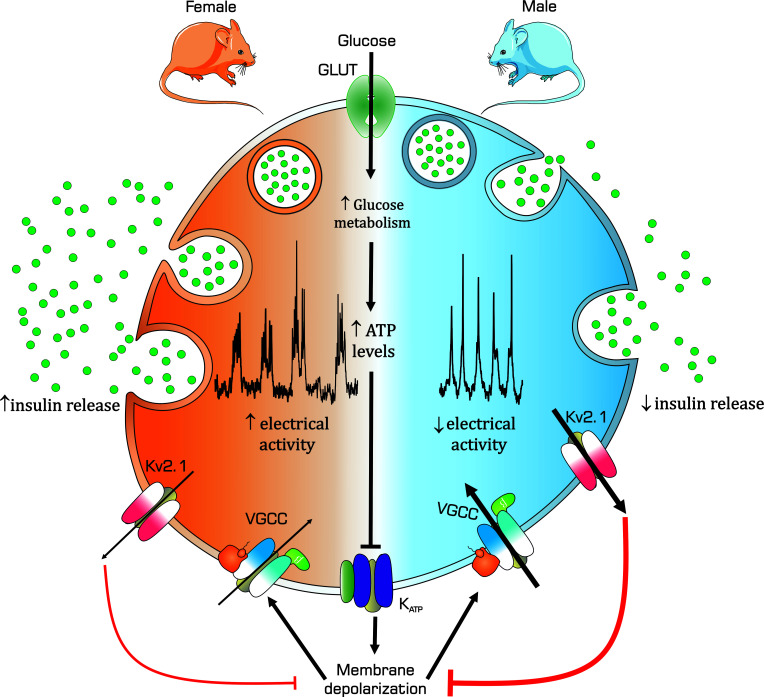

## Introduction

Type 2 diabetes mellitus (T2DM) is a heterogeneous disease characterized by chronic high blood glucose levels caused by impaired insulin release or its effects. In humans, T2DM has a higher incidence in males compared with females (1–4), a phenotype recapitulated by many rodent models (5–9). While different insulin sensitivity can account for this phenomenon, we hypothesize that hitherto uncharacterized sex differences in pancreatic β cell insulin release are also a critical contributor. β Cells of mice and men express a plethora of ion channels that control the glucose-induced electrical activity and insulin vesicle exocytosis (10–14). The glucose-dependent depolarization of the membrane potential (MP) is inversely controlled by the activity of ATP-sensitive K^+^ channels (K_ATP_) (15, 16). β Cell glucose uptake and metabolism increase the cytosolic ATP/ADP ratio that almost completely blocks the K_ATP_ channels in extracellular glucose levels higher than 10 mM (17). However, even when the K_ATP_ channels are completely blocked, the β cell MP remains below –40 mV, owing to the activity of background TASK-1 (18) and TALK-1 (19) 2-pore K^+^ channels (K_2_P). The glucose-dependent MP depolarization activates the high-voltage-gated Ca^2+^ channels (Ca_V_) (14, 20, 21). The Ca_V_ Ca^2+^ influx leads to an increase in intracellular Ca^2+^ concentration that locally triggers insulin vesicle exocytosis (11, 22–27). Concomitantly, Ca^2+^ influx through high-voltage-gated Ca^2+^ channels generates the characteristic Ca_V_-dependent β cell electrical activity (12, 14, 28, 29). The glucose-induced electrical activity of the β cells consists of trains of action potentials (APs) superimposed on a depolarizing plateau potential (PP) separated by hyperpolarized silent periods (30, 31). Increasing the extracellular glucose concentration prolongs the AP-train duration, culminating with a continuous firing in glucose concentrations above 15 mM. During the AP train, the firing pattern can be characterized by single APs or bursts of APs (32). The membrane repolarization during an AP and at the end of an AP train is supported by the activation of large-conductance (BK) (33, 34) and small-conductance (SK) Ca^2+^-activated K^+^ channels (35, 36). However, in both mouse (32) and human (37, 38) β cells, the membrane repolarization after an AP is primarily controlled by the activation of the voltage-gated K_v_2.1 K^+^ channels (39). K_v_2.1 genetic ablation (32) or pharmacological inhibition (37, 40–43) prolongs AP duration and increases the percentage of cells firing AP bursts. This results in increased cytosolic Ca^2+^ concentration, causing higher insulin release and enhanced glucose tolerance (32, 41, 42). Additionally, K_v_2.1 can directly increase insulin release independently of its effects on β cell electrical activity by enhancing vesicle recruitment and exocytosis (44–46) as well as promoting β cell survival (43, 47).

Previously, we have shown that female mice have enhanced glucose tolerance compared with males (48). However, this was not caused by sex-specific differences in insulin sensitivity at 4 weeks or pancreatic islet area at 14 weeks. Paradoxically, despite similar β cell Ca_V_ Ca^2+^ current influx, the glucose-induced islet Ca^2+^ transients were smaller in females compared with males, but caused a significantly higher second-phase insulin release (48).

Here, we aimed to determine whether the higher insulin release from female islets is caused by an enhanced glucose-induced electrical activity, higher intracellular Ca^2+^ amplification, or enhanced insulin granule exocytosis. Mechanistically, our data show that glucose stimulation leads to a higher membrane depolarization and electrical activity in female β cells compared with those of males. The more depolarized MP in female β cells led to reduced Ca_V_ channel availability, explaining the previously observed smaller glucose-induced islet Ca^2+^ transients (48). The sex-specific difference in β cell MP was caused by approximately 2-fold higher K_v_2.1 K^+^ currents in male compared with female β cells. Despite a different K_v_2.1 conductance, the insulin granule exocytosis was identical between sexes. Therefore, the smaller K_v_2.1 K^+^ currents promote glucose-induced electrical activity, leading to a larger second-phase insulin release and better glucose tolerance in females.

## Results

### Female β cells have higher electrical activity compared with those of males.

**Figure 1:**
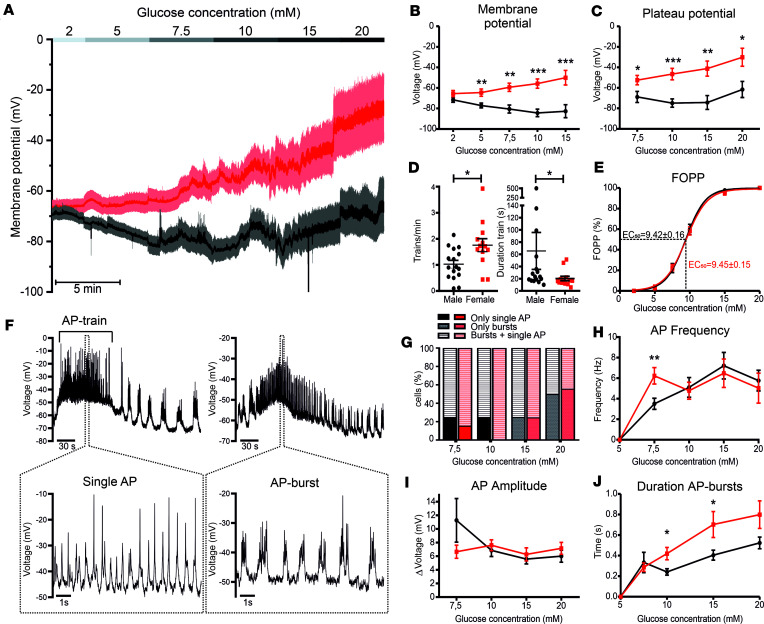
Female β cells show a more depolarized MP and PP and higher electrical activity in all stimulatory glucose concentrations compared with male β cells. (**A**) Average traces of the glucose-induced electrical activity in male (black, *n* = 10–20; 8 mice) and female β cells (red, *n* = 11–14; 6 mice) stimulated with 2, 5, 7.5, 10, 15, and 20 mM glucose. Female β cells show a significantly more depolarized MP (**B**) and PP (**C**). (**D**) AP-train frequency in 10 mM glucose was significantly higher in female β cells, while the duration of the AP trains was significantly longer in males. This leads to a similar fraction of plateau phase (FOPP) and glucose sensitivity (EC_50_) (**E**). (**F**) Sample traces of characteristic β cell electrical activity showing single-AP-firing mode (left) and AP-burst mode (right). (**G**) Percentage of cells showing the different firing patterns induced by different glucose concentrations from male (black) and female (red) β cells. Single AP firing is shown in dark colors, AP burst is shown in gray and light red, while mixed firing is represented by the striped pattern. (**H**) Frequency and (**I**) amplitude of APs in male and female β cells in different glucose concentrations. (**J**) AP-burst duration is longer in females in 10 and 15 mM glucose. All values are mean ± SEM. *P < 0.05, **P < 0.01, ***P < 0.001 by 2-tailed Student’s *t* test.

To characterize the glucose-induced electrical activity, we performed patch-clamp experiments in current-clamp mode in β cells still part of the intact pancreatic islet perfused with increasing glucose concentrations in a stepwise manner (2, 5, 7.5, 10, 15, and 20 mM) (Figure 1A). In 2 mM glucose, β cells of both sexes showed no electrical activity and a similar resting MP (RMP_females_= –65.6 ± 3.1 mV, RMP_males_= –71.7 ± 2.3 mV; Figure 1, A and B). Increasing the glucose concentration led to an increase in the electrical activity in a concentration-dependent manner in β cells of both sexes. However, the average of the electrical activity shows that only female β cells responded with a steady membrane depolarization (Figure 1A). Sample traces (Supplemental Figure 1; supplemental material available online with this article; https://doi.org/10.1172/jci.insight.171609DS1) show that the MP of male β cells frequently undergoes episodes of hyperpolarization, while in female β cells the MP remains depolarized. Therefore, the average MP will be more hyperpolarized in males compared with female β cells (Figure 1A and Supplemental Figure 1). The most negative MPs (Figure 1B) and PP (Figure 1C) were significantly more depolarized in females compared with males (i.e., in 15 mM glucose, MP_females_ = –50.0 ± 7.1 mV, MP_males_ = –82.7 ± 6.3 mV, *P* < 0.001; PP_females_ = –41.22 ± 7.35 mV, PP_males_ = –74.3 ± 6.8 mV; *P* = 0.003). In addition, the AP-train frequency (f) in 10 mM glucose was significantly higher in female compared with male β cells (f_females_ = 1.76 ± 0.25 trains/min, f_males_ = 1.14 ± 0.15 trains/min; *P* = 0.03) (Figure 1D). Despite the higher AP-train frequency, the fraction of time that a β cell spent in the PP phase (FOPP) at each stimulatory glucose concentration was identical between sexes, demonstrating that the β cells of both sexes have a similar glucose sensitivity (EC_50_ glucose_female_ = 9.45 ± 0.15 mM, EC_50_ glucose_male_ = 9.42 ± 0.16 mM) (Figure 1E). This apparent discrepancy can be reconciled by the observation that in 10 mM glucose, male β cells displayed longer AP trains compared with females (Duration-train_male_ = 65.5 ± 30.2 seconds, Duration-train_female_ = 20.3 ± 3.7 seconds; *P* = 0.03) (Figure 1D). The AP firing properties showed many sex-specific differences (Figure 1, F–J). During an AP train, β cells can display 2 different firing patterns: single AP firing and AP bursts (Figure 1F). In 7.5 and 10 mM extracellular glucose, females had a higher percentage of cells showing burst firing mode compared with males. In 10 mM glucose, 25% of the cells from males show only single AP firing, while in females 100% of the cells showed a mixed firing pattern of both single APs and AP bursts (Figure 1G). While the AP or AP-burst frequency and amplitude (Figure 1I) were not significantly different between sexes in most glucose concentrations, the AP frequency in 7.5 mM glucose (Figure 1H) (f_males_ = 3.5 ± 0.5 Hz, f_females_ = 6.2 ± 0.8 Hz; *P* = 0.0086) and the AP-burst duration (Figure 1J) were almost double in females compared with males (in 10 mM glucose, Burst-duration_females_ = 0.42 ± 0.06 seconds and Burst-duration_males_ = 0.24 ± 0.03 seconds; *P* = 0.04). These results demonstrate that the glucose-induced electrical activity is higher in females compared with males as a result of a higher frequency of AP trains and higher AP firing frequency at lower glucose concentration, higher incidence of AP bursts, and longer AP-burst duration.

### K_ATP_ and K_2_P currents do not differ between sexes.

**Figure 2:**
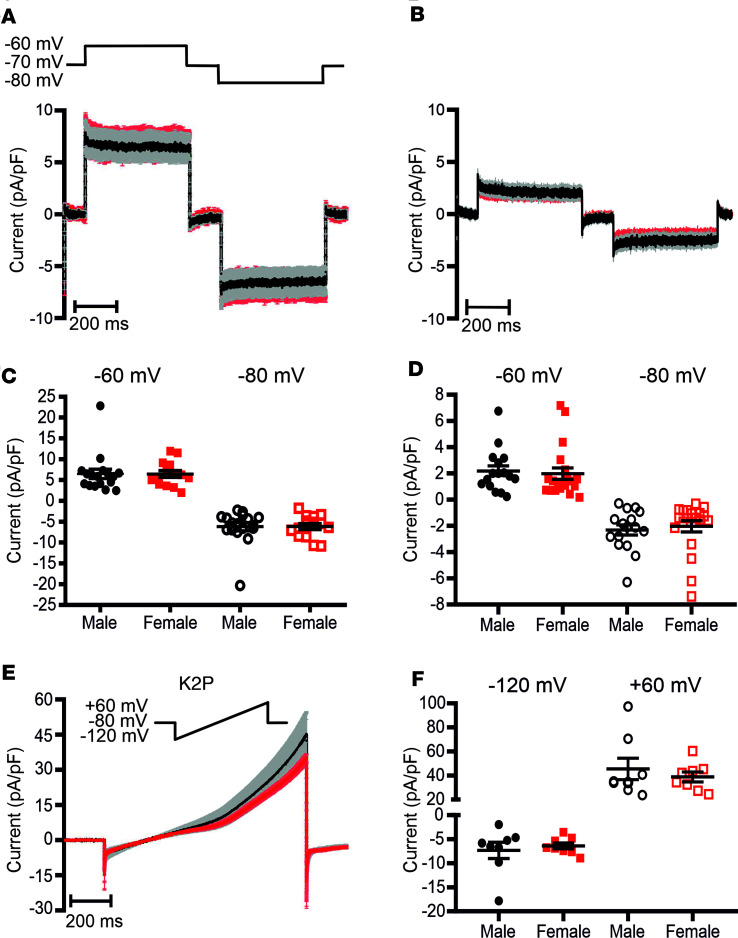
K_ATP_ and TASK/TALK currents do not differ between β cells of both sexes. (**A**) Average traces of males (black, *n* = 17; 3 mice) and female (red, *n* = 15; 3 mice) K_ATP_ current in the presence of 2 mM glucose and 340 mM diazoxide (Dzx). (**B**) K_ATP_ currents in the presence of 5 mM extracellular glucose. (**C**) K_ATP_ current density at –60 mV (filled symbols) and –80 mV (empty symbols) in the presence of 2 mM glucose plus 340 mM Dzx. (**D**) K_ATP_ current density at –60 mV (filled symbols) and –80 mV (empty symbols) in the presence of 5 mM glucose. (**E**) One-second ramp protocol used to record the K_2_P currents from –120 mV to +60 mV (top) and average traces of K_2_P current recorded in male (black, *n* = 8; 3 mice) and female (red, *n* = 8; 3 mice) β cells. (**F**) K_2_P current densities at –120 mV (filled symbols) and at +60 mV (empty symbols) in β cells of both sexes. All values are mean ± SEM. Significance was evaluated by 2-tailed Student’s *t* test.

K_ATP_ channel activity is responsible for maintaining the pancreatic β cells’ hyperpolarized RMP under low extracellular glucose levels (15, 16). To investigate whether the higher glucose-induced membrane depolarization in female β cells is caused by different K_ATP_ channel expression or activation, we measured the K^+^ currents (IK) using a ±10 mV square pulse protocol from a holding potential of –70 mV (Figure 2A). The average traces of K_ATP_ current densities in the presence of 2 mM extracellular glucose and 340 μM diazoxide, a K_ATP_ channel activator that works independently of the ATP/ADP ratio, showed no sex difference (i.e., in 2 mM glucose and at –60 mV IK_ATP-male_ = 6.47 ± 1.12 pA/pF, IK_ATP-female_ = 6.45 ± 0.8 pA/pF; *P* = 0.98) (Figure 2, A and C). Considering that in 5 mM glucose the β cell MP was significantly different between sexes (Figure 1B), we asked whether the K_ATP_ channel endogenous inhibition is sex specific. When measured in 5 mM glucose, the K_ATP_ currents were also similar in the β cells of both sexes at both tested MPs (i.e., in 5 mM glucose and at –60 mV IK_ATP-male_ = 2.17 ± 0.41 pA/pF, IK_ATP-female_ = 1.98 ± 0.43 pA/pF; *P* = 0.74) (Figure 2, B and D). Together, these results demonstrate that the K_ATP_ channel expression or inhibition in pancreatic β cells is not sex specific. Other potential targets involved in maintaining the MP and PP in high glucose concentrations are the K_2_P channels TASK-1 and TALK-1 (18, 19). However, a 1-second-long ramp protocol from –120 mV to +60 mV (Figure 2E) elicited similar K^+^ current densities in β cells of both sexes (i.e., at –120 mV IK_2_P_male_ = –7.32 ± 1.7 pA/pF; IK_2_P_female_ = –6.41 ± 0.61 pA/pF) (Figure 2, E and F), thus excluding their involvement in the sex-specific β cell MP.

### β Cell–induced electrical activity is similar between sexes.

**Figure 3:**
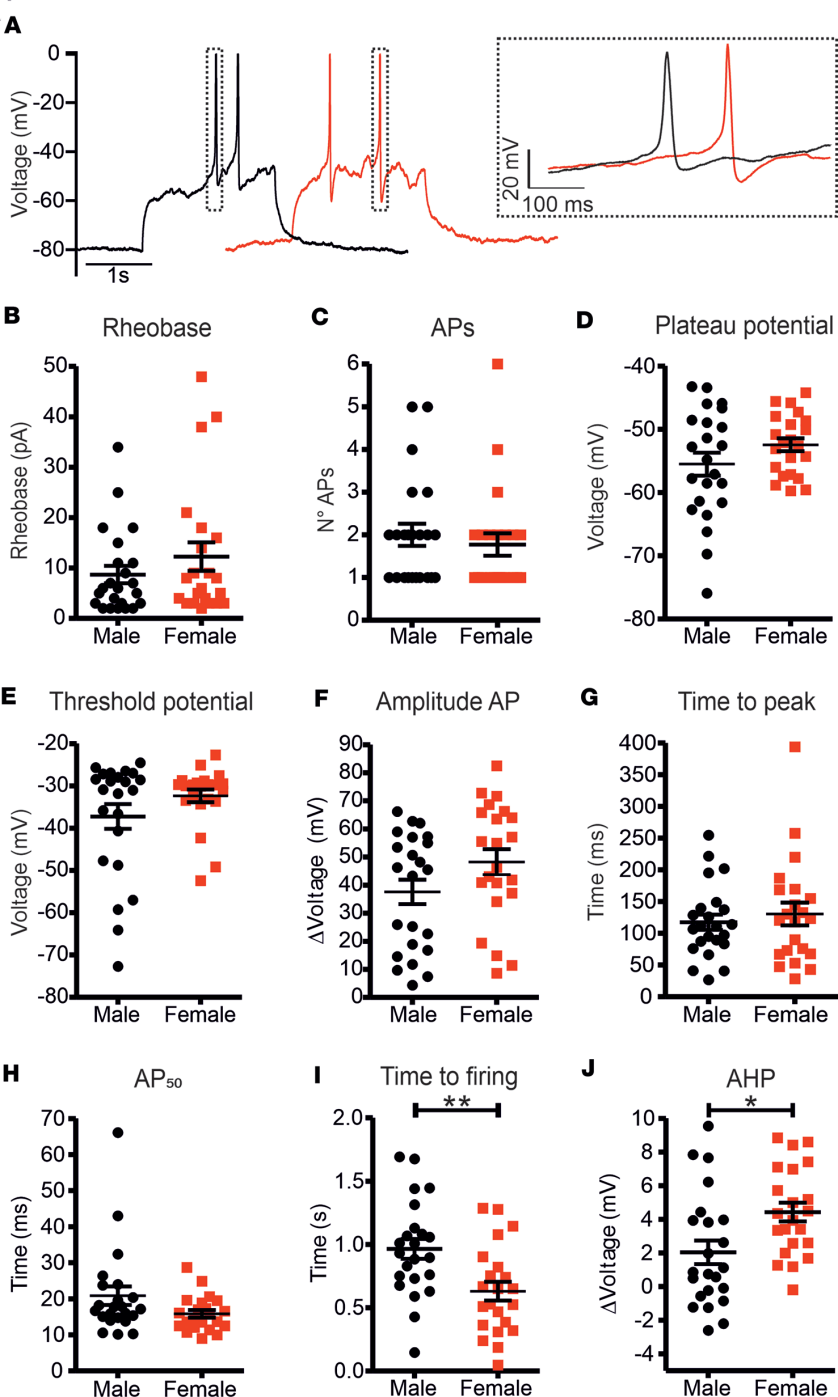
Induced electrical activity in isolated β cells from male and female mice. (**A**) Sample traces of the induced electrical activity recorded at the rheobase current injection from both male (black, *n* = 23; 3 mice) and female (red, *n* = 21; 3 mice) isolated β cells. The rheobase was not significantly different between males and females (**B**) and neither was the number of APs (**C**). Both the plateau potential (**D**) and the threshold potential of the AP upstroke phase (**E**) were not significantly different, nor were the AP amplitude (**F**), time to peak (**G**), and 50% of the maximal amplitude (AP_50_) (**H**). The time to firing of the first AP (**I**) was significantly longer in male β cells, while the AP after hyperpolarization (AHP) component (**J**) was significantly higher in female β cells compared with males. All the parameters shown are calculated from the first AP at the rheobase. All values are mean ± SEM. *P < 0.05, **P < 0.01 by 2-tailed Student’s *t* test.

The different MPs alone could be responsible for all observed sex-specific differences in AP frequency and AP-burst duration (Figure 1). To test this hypothesis, we measured the induced electrical activity in response to stepped-current injections in single isolated pancreatic β cells. For this, we clamped the β cell MP at –80 mV and induced depolarizations using 2-second-long current injections in 2-pA increments (Figure 3A). The minimal current injected to elicit AP firing, also referred as rheobase, was similar between β cells of both sexes (Figure 3B). Similarly, the number of APs recorded at rheobase, the PP and AP threshold potential, as well as AP amplitude, time to peak, and the 50% of the maximal AP amplitude did not show any sex difference (Figure 3, C–H). These observations demonstrate that the sex difference in AP frequency and AP-burst duration were largely caused by the altered MP. However, male β cells take significantly longer time to reach the AP depolarization threshold (t_males_= 0.97 ± 0.08 seconds, t_females_ = 0.63 ± 0.07 seconds; *P* = 0.0035) (Figure 3I) and the after-hyperpolarization (AHP) amplitude was larger in females compared with males (AHP_males_ = 2.04 ± 0.70 mV, AHP_females_ = 4.43 ± 0.56 mV; *P* = 0.0126) (Figure 3J), indicating possible sex-specific differences in β cell voltage-gated (K_v_) and Ca^2+^-activated K^+^ currents.

### The Ca^2+^-activated K^+^ current amplitudes are similar between sexes but show different kinetics and Ca^2+^ dependence.

**Figure 4:**
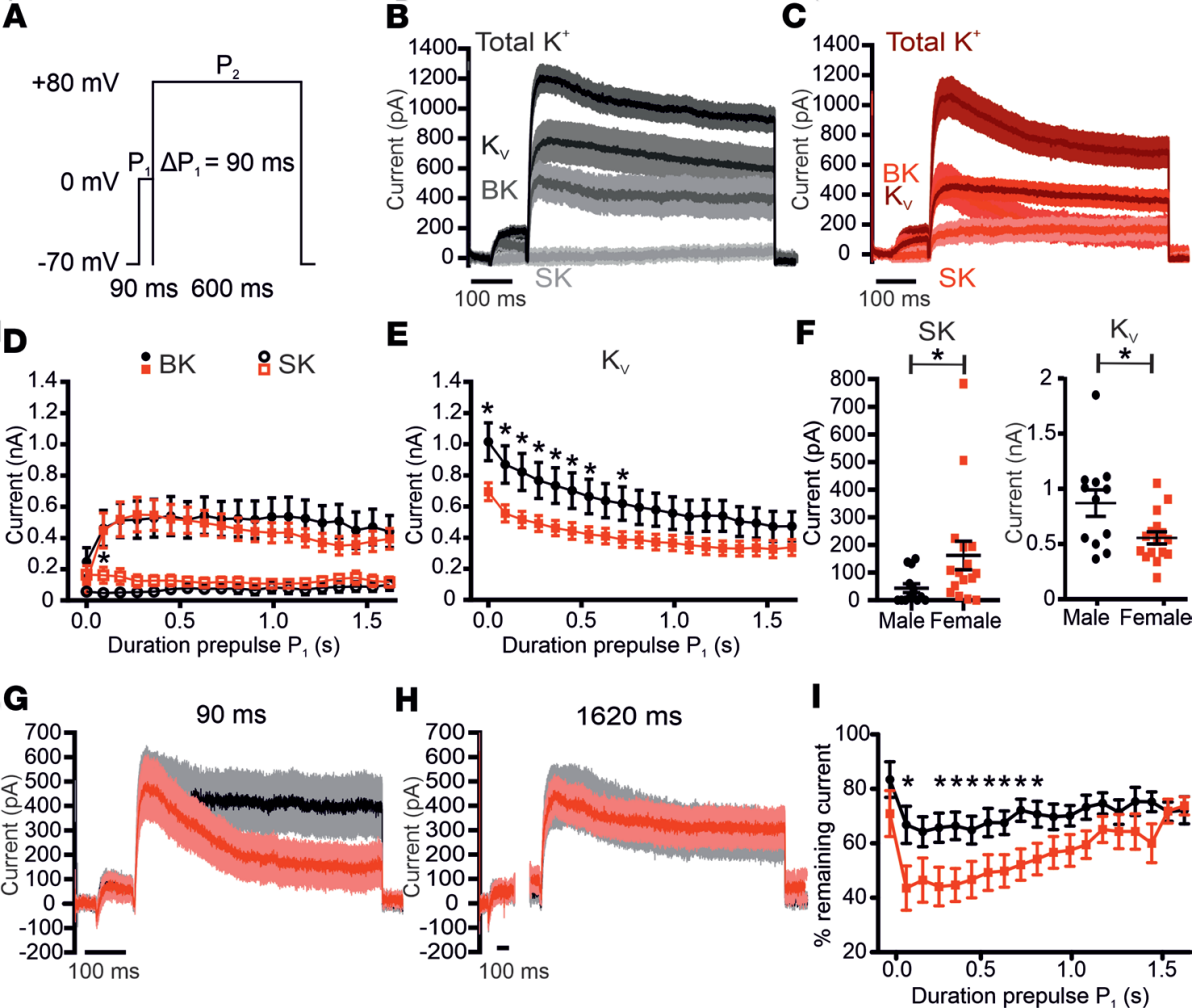
Ca^2+^-activated K^+^ current amplitude is similar in β cells of both sexes but shows different kinetics and Ca^2+^ dependence. (**A**) Protocol used for measuring Ca^2+^-activated K^+^ current, which consisted of a variable-length Ca^2+^-loading prepulse to 0 mV (P_1_), with each sweep increasing in length by 90 ms, followed by a test pulse (P_2_) to +80 mV to measure K^+^ currents. (**B** and **C**) Average trace of total, BK, SK, and K_v_ currents from male (*n* = 12; 3 mice) (**B**) and female (*n* = 16; 4 mice) (**C**) β cells at P_1_ = 90 ms. (**D**) The BK (full symbols) and SK (empty symbols) current components were obtained by subtraction from the total K^+^ current after the sequential addition of 1 μM paxilline and 200 nM apamin. (**E**) The remaining current after BK and SK block represents the K_v_ current component. (**F**) Scatter plot showing the significantly higher SK (left) and K_V_ currents (right) at P_1_ = 90 ms. (**G**) The BK current kinetics following a P_1_ = 90 ms or (**H**) P_1_ = 1620 ms prepulse recorded in male and female β cells. (**I**) The remaining BK current at the end of the 600 ms P_2_ pulse is significantly larger in male β cells compared with females if the P_1_ prepulse is shorter than 800 ms. All values are mean ± SEM. *P < 0.05 by Mann-Whitney test (**D** and **F**) or 2-tailed Student’s *t* test (**E**, **F**, and **I**).

In pancreatic β cells, Ca^2+^-activated BK and SK K^+^ channels contribute to AP repolarization and shape (34, 49–54). To characterize the BK and SK channel Ca^2+^ dependence, we used a double-pulse protocol consisting of a variable-length prepulse to 0 mV in 90-ms increments that opens the Ca_V_s, thus progressively loading the cell with Ca^2+^, followed by a test pulse to +80 mV that activates the BK and SK currents (Figure 4A). The BK and SK components were isolated by subtracting the currents in the presence of 1 μM paxilline and 200 nM apamin from the total K^+^ currents. The maximal BK current amplitudes (Figure 4D) did not differ between male (Figure 4B) and female β cells (Figure 4C). However, the BK current decay was significantly faster in females if the Ca^2+^ loading prepulse was shorter than 0.8 seconds (Figure 4, G–I). Following a Ca^2+^ loading prepulse of 90 ms, the remaining current after a 500-ms test pulse was approximately 1.5-fold higher in males compared with females (I_BK_-R_500male_ = 66.8% ± 6.81%, I_BK_-R_500female_ = 43.55% ± 8.12%; *P* = 0.04) (Figure 4, G and I). After the application of 200 nM apamin, the calculated SK current component was significantly higher in females compared with males only if the Ca^2+^ preloading step was 90 ms long (I_SKmale_ = 47.08 ± 17.06, I_SKfemale_ = 161.88 ± 51.67; *P* = 0.037) (Figure 4, D and F). Together, these results demonstrate a different contribution of BK and SK K^+^ currents to β cell membrane repolarization during an AP at low-frequency electrical activity, explaining the observed higher AHP component in female β cells compared with males (Figure 3J). However, during the repetitive firing of an AP train, simulated in our protocol by longer Ca^2+^ preloading steps, the contribution of BK and SK to β cell electrical activity will be similar in both sexes. Interestingly, the remaining voltage-gated K^+^ currents after the addition of both 1 μM paxilline and 200 nM apamin were significantly higher in males compared with females (IK_Vmales_ = 1015.25 ± 121.59 pA, IK_Vfemales_ = 694.25 ± 58.07 pA; *P* = 0.016) (Figure 4, E and F).

### Male β cells have larger K_v_2.1 currents that set the MP.

**Figure 5:**
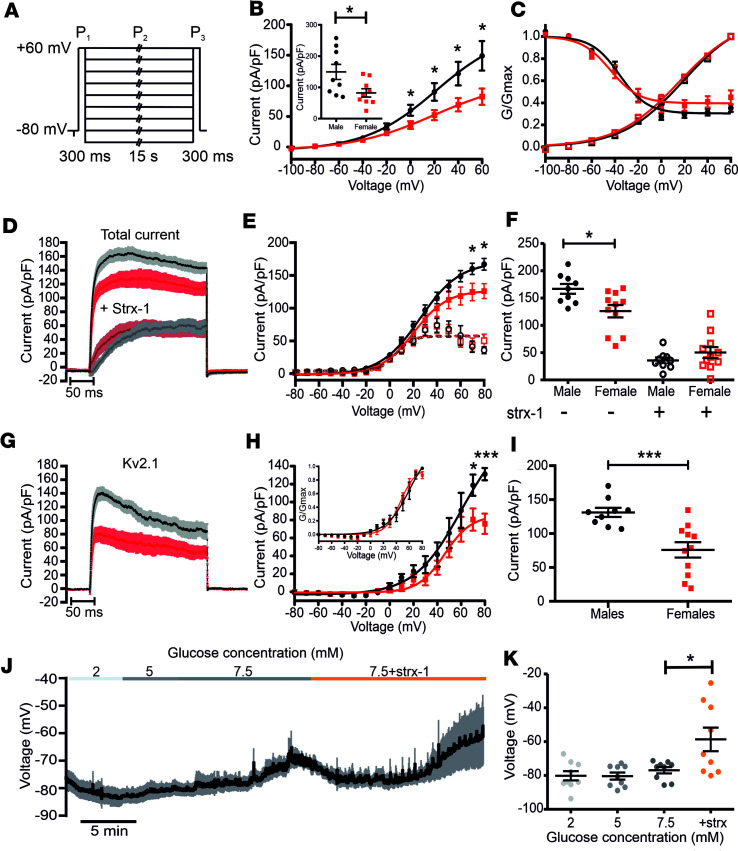
K_v_2.1 current density is higher in males compared with females and it modulates the MP. (**A**) The 3-pulse protocol used to characterize the voltage sensitivity of the K_v_ channel activation and inactivation. (**B**) The current density amplitude was significantly higher in males (black, *n* = 9; 3 mice) compared with females (red, *n* = 9; 3 mice). Inset showing the scatter plot of the K_v_ current density at +60 mV. (**C**) The K_v_ current voltage dependence of activation (empty symbols) and inactivation (full symbols) were not different between β cells of male and female mice. (**D**) Average trace of the K^+^ current measured in males (*n* = 9; 3 mice) and female (*n* = 9; 3 mice) in the absence or presence of 100 nM specific K_v_2.1 blocker stromatoxin-1 (Strx-1) (males in gray; females in wine). (**E**) K^+^ current-voltage relationship without Strx-1 (full symbols) and after the addition of Strx-1 (empty symbols) in pancreatic β cells of both sexes. (**F**) Scatter plot showing the individual values of the K^+^ currents with and without Strx-1 in both male and female β cells at +80 mV. (**G**) Average trace of Strx-1–sensitive K_v_2.1 K^+^ currents calculated by subtraction from panel **D**. (**H**) Current-voltage relationship of K_v_2.1 currents. Inset shows the normalized conductance. (**I**) Scatter plot of the K_v_2.1 current density at +80 mV. (**J**) Average of glucose-induced electrical activity of male β cells (*n* = 9; 3 mice) in intact islets with and without 100 nM Strx-1. (**K**) Scatter plot of the the average MP in male β cells with and without 100 nM Strx-1. All values are mean ± SEM. *P < 0.05; ***P < 0.001 by paired or unpaired, 2-tailed Student’s *t* test.

To investigate whether this observed change in the K^+^ current amplitude is also associated with a sex-specific modulation of K_v_ channel voltage dependence of activation and inactivation, we used a 3-pulse protocol (55) consisting of 2 test pulses 300 ms long to +60 mV (P_1_ and P_3_) separated by a 15-second-long conditioning pulse (P_2_) from –100 mV to +60 mV in 20-mV increments (Figure 5A). The voltage dependence of activation was determined using the maximal amplitude during the conditioning P_2_ pulse, while the voltage dependence of inactivation was measured using the maximal amplitude of the last P_3_ test pulse in comparison with the P_1_ pulse. The conditioning pulse elicited significantly higher K^+^ currents in male β cells compared with females at membrane depolarization above 0 mV (at +60 mV, IK_Vmales_ = 149 ± 24.1 pA/pF; IK_Vfemales_ = 82.3 ± 13.3 pA/pF; *P* = 0.0269) (Figure 5B). Nevertheless, the voltage dependence of activation and inactivation (Figure 5C) as well as the percentage of inactivation (data not shown) remained similar between β cells of both sexes. β Cells express several K_v_ channel isoforms, with K_v_2.1 conducting approximately 70% of the total K_v_ currents (32, 37, 38, 56). To investigate whether the observed sex difference in K_v_ current amplitude is caused by different K_v_2.1 conductance, we measured the total K^+^ current in the absence and presence of the specific K_v_2.1 blocker stromatoxin-1 (Strx-1, 100 nM) using a 500-ms-step depolarization pulse from –80 mV to +80 mV in 10-mV increments (Figure 5, D and G). Male β cells had significantly higher total K^+^ current density (Figure 5, E and F) compared with females (IK_Vmales_ = 166.97 ± 8.97 pA/pF, IK_Vfemales_ = 126.1 ± 11.3 pA/pF; *P* = 0.0135). The remaining K^+^ current after the addition of 100 nM Strx-1 was similar in β cells of both sexes, demonstrating that only K_v_2.1 conductance is sex specific (Figure 5, E and F). Calculating the Strx-1–sensitive component by subtraction showed that male β cells conduct approximately 1.7-fold higher K_v_2.1 currents compared with females (IK_v_2.1_males_ = 131.15 ± 6.79 pA/pF, IK_v_2.1_females_ = 75.8 ± 11.34 pA/pF; *P* < 0.001) (Figure 5, H and I), with a similar voltage dependence of activation (Figure 5H, inset). Together, these results suggest that the different K_v_2.1 conductance accounts for the observed sex-specific difference in MP. However, this is in divergence with the known role of K_v_2.1 channels as delayed rectifier channels responsible for membrane repolarization during an AP. To investigate whether the MP is modulated by K_v_2.1, we measured the glucose-induced electrical activity in male β cells without any drug application and subsequently in the presence of 100 nM Strx-1 (Figure 5J). As shown in Figure 1, increasing the glucose concentration in male β cells from 2 to 5 and 7.5 mM did not result in an MP depolarization even after a continuous 30-minute application (Supplemental Figure 1). Nevertheless, the addition of 100 nM Strx-1 significantly depolarized the MP by approximately 20 mV (Figure 5, J and K, and Supplemental Figure 1). These results demonstrate what we believe is a novel role for K_v_2.1 in maintaining the β cells’ MP. Additionally, a different K_v_2.1 expression or localization could lead to a sex-specific insulin release independently of its ion-conducting pore function. Overexpression of a K_v_2.1 pore mutant channel increased vesicle exocytosis (44), while K_v_2.1 silencing resulted in impaired exocytosis due to reduced vesicle recruitment and fusion (45, 46). Therefore, a potentially reduced K_v_2.1 channel expression in female β cells might result in impaired vesicle exocytosis. Conversely, our previous report showing a higher insulin secretion and better glucose tolerance in females (48) might indicate more exocytosis from female β cells.

### Vesicle exocytosis is similar in β cells of both sexes, but the incretin pathway is different.

**Figure 6:**
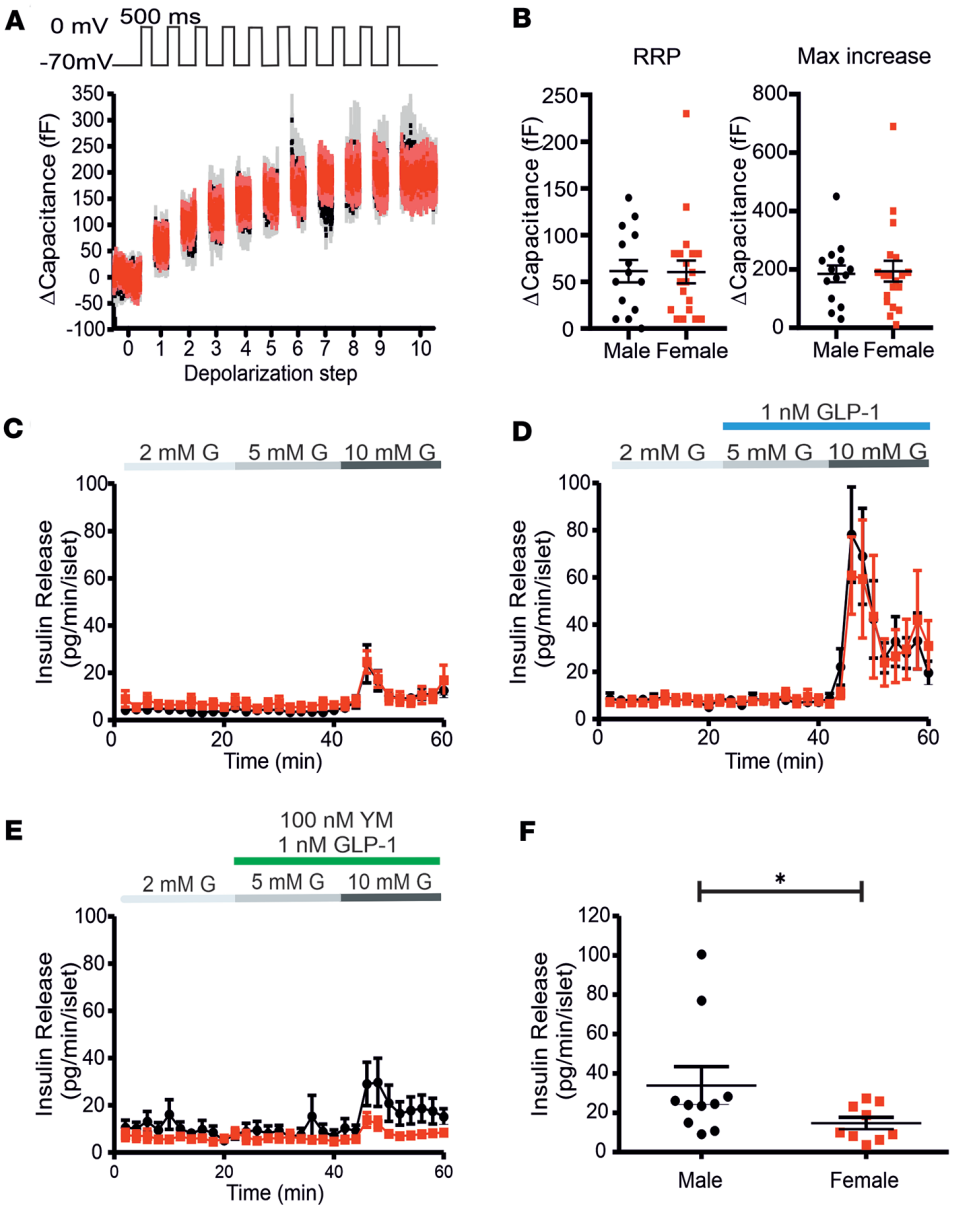
Vesicle exocytosis is similar in male and female β cells, but the incretin pathway shows a sex difference. (**A**) Average trace of the β cell capacitance increase after a train of 10 depolarizing steps (500-ms steps from –70 mV to 0 mV) from male (black, *n* = 15; 3 mice) and female (red, *n* = 19; 3 mice) mice. (**B**) Scatter plot of the increase in capacitance after the first depolarization, corresponding to the readily releasable pool (RRP, left) and the maximal exocytosis (right). (**C**) Dynamic insulin release from male (*n* = 10) and female (*n* = 9) islets (20 islets/experiment) in 2, 5, and 10 mM glucose (**G**). (**D**) Same as in **A** but in the presence of 1 nM GLP-1. (**E**) Dynamic insulin release in 2, 5, and 10 mM glucose plus 1 nM GLP-1 and 100 nM G_q_ inhibitor YM-254890. (**F**) Scatter plot of the peak insulin release in the presence of 10 mM glucose, 1 nM GLP-1, and 100 nM YM-254890. All values are mean ± SEM. *P < 0.05 by 2-tailed Student’s *t* test or Mann-Whitney test (**F**).

Insulin vesicle fusion with the plasma membrane increases the cell surface that can be measured as an increase in cell capacitance (Cm). In response to a train of 10 depolarizing steps 500 ms long to 0 mV that activates the Ca_V_ Ca^2+^ influx, the total increase in Cm was identical between β cells of both sexes (Figure 6, A and B). Following the first depolarization step, the initial Cm increase is a direct measurement of the already primed, readily releasable pool (RRP) of vesicles (Figure 6B). The β cells of both sexes did not show any difference either in the first or in the subsequent steps (Figure 6, A and B). Previously, it has been demonstrated that K_v_2.1 pharmacological inhibition (37, 42, 43) or genetic ablation (32) enhanced both phases of insulin release (42). However, consistent with our previous report (48) and the Cm measurements, the first phase of insulin release did not show any significant sex-specific difference (Figure 6C). Partial K_v_2.1 current inhibition has also been shown to enhance glucagon-like peptide-1 (GLP-1) receptor activation insulinotropic effects, but only at subthreshold exendin-4 levels and not maximal activation (42). Consistent with this observation, maximal receptor activation with 1 nM GLP-1 resulted in the same augmentation of insulin release in β cells of both sexes (Figure 6D). Nevertheless, recently it has been reported that chronically depolarized β cell MP alters the GLP-1 receptor downstream signaling cascade, showing a higher contribution of the G_q_ pathway compared with G_s_ (57). Consistent with this notion and the more depolarized MP in female β cells (Figure 1), the application of 100 nM YM-254890, a G_q_ pathway inhibitor, in parallel with 1 nM GLP-1 resulted in a significantly greater inhibition of insulin release from female islets compared with male islets (Insulin peak_males_= 33.9 ± 9.5 pg/min/islet, Insulin peak_females_= 14.75 ± 3.03 pg/min/islet; *P* = 0.04) (Figure 6, E and F). Importantly, the application of 100 nM YM-254890 alone in the absence of GLP-1 stimulation led to a similar, nonsignificant reduction in peak insulin release in islets of both sexes (Supplemental Figure 2), demonstrating that the basal G_q_ pathway activation does not show a sex difference. Together, these results demonstrate that neither insulin vesicle priming and release nor maximal incretin effects are sex specific. This indicates that the previously reported higher second-phase insulin release in female islets could be caused by the increased electrical activity alone. However, contradicting this notion is the observation that the glucose-induced islet Ca^2+^ transients were smaller in females compared with males, despite similar Ca_V_ Ca^2+^ current density (48).

### Different MP alters the Ca_V_ channel availability.

**Figure 7:**
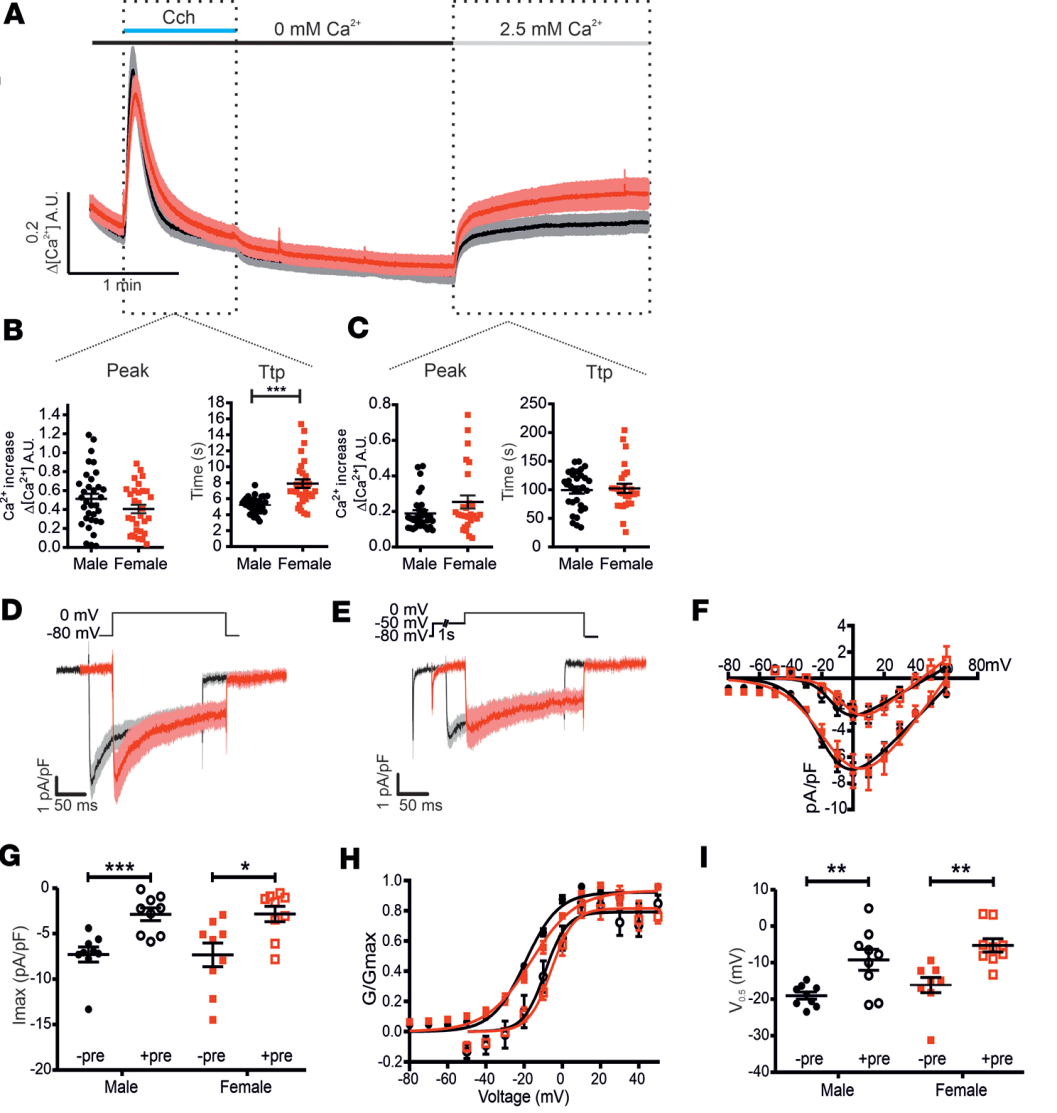
Increased MP reduces the HVCC availability. (**A**) Average Ca^2+^ transients in islets of male (black, *n* = 31–33; 3 mice) and female mice (red, *n* = 26–30; 3 mice). Cch, carbachol. (**B**) Peak amplitude and time to peak (TTP) of Ca^2+^-store release induced by 0.2 mM carbachol in the presence of 2.5 mM extracellular Ca^2+^. (**C**) SOCE Ca^2+^ transients measured upon perfusion with 2.5 mM Ca^2+^–containing solution. (**D**) Average trace of the Ca^2+^ influx in male (black, *n* = 9; 3 mice) and female (red, *n* = 9; 3 mice) β cells from a holding potential of –80 mV or (**E**) after a 1-second-long prepulse to –50 mV. (**F**) The I/V curve and scatter plot of the maximum Ca^2+^ current density (**G**) showing that the prepulse to –50 mV induces a reduction in the Ca^2+^ influx by approximately 60% in both male and female β cells. (**H**) The prepulse to –50 mV shifts the voltage dependence of Ca^2+^ conductance by 10 mV toward more depolarized potentials. (**I**) Scatter plot of half maximal activation values (V_0.5_). All values are mean ± SEM. *P < 0.05; **P < 0.01; ***P < 0.001 by 2-tailed Student’s *t* test.

The smaller islet Ca^2+^ transients despite similar Ca^2+^ influx could be a consequence of reduced intracellular Ca^2+^-store filling and release. To test this hypothesis, we measured the endoplasmic reticulum (ER) intracellular store Ca^2+^ release by activation of the inositol 1,4,5-trisphosphate (IP_3_) receptors (IP_3_Rs) with 200 nM carbachol (Cch) (Figure 7A). To exclude the contribution of Ca^2+^ influx to cytosolic Ca^2+^ concentration, the islets were bathed in an extracellular solution containing 0 mM Ca^2+^. The peak amplitude of ER Ca^2+^ release had the same amplitude in islets of both sexes (Figure 7, A and B); however, the time to peak of Ca^2+^ transients in female islets was significantly slower (CchTtp_male_ = 5.21 ± 0.17 seconds, CchTtp_females_ = 7.89 ± 0.52 seconds; *P* < 0.001). ER-store depletion activates the β cell store–operated Ca^2+^ entry (SOCE) (58, 59). Following perfusion with physiological solution containing 2.5 mM Ca^2+^, the islets of both sexes showed similar SOCE amplitude and kinetics (Figure 7C). If Ca_V_ Ca^2+^ influx density (48), intracellular Ca^2+^ store release, and ER-depletion-triggered SOCE are similar, then how could the islet Ca^2+^ transients be reduced in female islets (48) compared with males? Upon membrane depolarization, most Ca_V_ channel isoforms undergo time-dependent conformational rearrangements that drive the channel into an inactivated state (14). To test the effect of the sex-specific difference in MP (Figure 1B) on Ca_V_ availability, we measured the Ca^2+^ current density in male and female β cells starting from an MP of –80 mV, corresponding to the male β cells (Figure 7D), or –50 mV (Figure 7E), corresponding to the measured MP in female β cells stimulated with 15 mM glucose (Figure 1B). Confirming our previous report (48), Ca^2+^ influx in β cells of both sexes had a similar amplitude and voltage dependence if the MP is identical (Figure 7, F–I). However, if the MP is maintained at –50 mV, then the Ca^2+^ current amplitude in β cells of both sexes decreases by approximately 60% (Figure 7, F and G) (Males: Imax _-80mV_ = –7.3 ± 0.83 pA/pF, Imax_–50mV_ = –2.88 ± 0.7 pA/pF; *P* < 0.001. Females: Imax_–80mV_ = –7.34 ± 1.3 pA/pF, Imax_–50mV_ = –2.84 ± 0.85 pA/pF; *P* = 0.011). Additionally, the voltage dependence of activation was shifted toward higher potentials by approximately 10 mV (Figure 7, H and I) (Males: Vhalf_–80mV_ = –19 ± 0.98 mV, Vhalf_–50mV_ = –9.2 ± 2.8 mV; *P* = 0.01. Females: Vhalf_–80mV_ = –16.09 ± 2.0 mV, Vhalf_–50mV_ = –5.3 ± 1.8 mV; *P* = 0.0012). Therefore, the increased MP in female β cells reduces the Ca_V_ channel availability, resulting in smaller Ca^2+^ influx compared with male β cells. Consequently, the reduced Ca^2+^ influx will lead to a smaller amplitude of glucose-induced Ca^2+^ transients in female islets compared with male islets.

## Discussion

In humans, the prevalence of diabetes shows a clear sexual dimorphism, with males being more affected compared with females (1–4, 60–65). While peripheral insulin sensitivity is a contributing factor, sex-specific differences in β cell insulin release could also be a strong determinant. Previously, we reported that following an intraperitoneal glucose tolerance test female mice have a significantly higher second-phase insulin release compared with males (48). Paradoxically, the glucose-induced Ca^2+^ transients were significantly smaller in females, while the Ca^2+^ current densities remained unaltered between sexes. Here, we show that a higher glucose-induced membrane depolarization and electrical activity is responsible for this phenomenon in female β cells compared with those of males. Surprisingly, the more depolarized MP in female β cells is not caused by sex-specific K_ATP_ or K_2_P channel activity, but significantly reduced K_V_2.1 channel conductance. Previously, it has been proposed that K^+^ efflux mediated by the K_V_2.1 delayed rectifier channel determines the AP repolarization phase (37, 39, 56, 66). Consequently, K_V_2.1 genetic ablation in pancreatic β cells increased the AP duration (32). In line with these observations, our data show that female β cells display longer AP duration manifested as an increase in the percentage of cells showing AP bursts as well as longer AP-burst duration. However, a contradictory finding seems to be that the reduced K_V_2.1 currents in female β cells stimulated with 7.5 mM extracellular glucose resulted in an increased AP frequency, but K_V_2.1 genetic deletion resulted in a reduced AP firing frequency (32). These K_V_2.1^–/–^ β cells showed a reduced membrane repolarization that is bound to decrease the Ca_V_ and Na_V_ channel availability, thus reducing the firing frequency (32). In our experiments, the K_V_2.1 current density was only partially reduced in female β cells, resulting in a stronger glucose-induced membrane depolarization that sets the PP in the range of the L-type Ca^2+^ channel activation threshold, thus increasing the firing frequency.

The higher electrical activity and reduced K_V_2.1 currents not only increase insulin release, but also promote β cell survival. K_V_2.1 overexpression in the INS-1 β cell line activated mitochondrial and ER stress–induced apoptosis (47). Consistent with this observation, it has been shown that K_V_2.1 pharmacological inhibition increases serum insulin levels, restores β cell mass, and decreases fasting blood glucose in a diabetes mouse model (43). The effects could be mediated via an enhanced electrical activity–dependent metabolic memory (67, 68) or alternatively via a reduced Ca^2+^ toxicity. Supporting this notion comes the observation that the more depolarized MP in female β cells causes a significantly reduced Ca_V_ channel availability and Ca^2+^ influx and therefore reduced glucose-induced islet Ca^2+^ transient amplitude (48). In contrast, the larger K_V_2.1 current in male β cells causes a hyperpolarized PP, leading to higher Ca_V_ channel availability and therefore increased amplitude of the glucose-induced islet Ca^2+^ transients. Supporting the Ca^2+^ toxicity hypothesis is also the observation that L-type Ca^2+^ channel pharmacological inhibition using verapamil delays β cell mass reduction in T1DM and T2DM diabetes patients or mouse models (69–72). In line with these concepts is also the observation that under ER stress conditions islets isolated from female mice showed a greater ability to maintain glucose-stimulated insulin production and secretion caused by higher expression of genes linked with protein synthesis, folding, and processing (60).

Besides controlling the firing frequency, our experiments show that the most important role of K_V_2.1 currents in male β cells is to maintain a hyperpolarized MP. The question arises as to how a delayed rectifier K^+^ channel that should be activated only during an AP can modulate the MP in the absence of the electrical activity. The MP in β cells is characterized by constant oscillations generated by the activation of background depolarizing and hyperpolarizing currents. Depending on the extracellular glucose concentration, the peak of the oscillations might be below the depolarization threshold of the Ca_V_ and Na_V_ channels and therefore would not elicit an AP. However, these oscillations could activate a very small percentage of K_v_2.1 channels. Based on the experimentally determined voltage dependence of activation and inactivation (Figure 5C), we calculate using Boltzmann equations that at –60 mV approximately 2% of K_v_2.1 current is activated. Considering the sex-specific K_v_2.1 maximal current densities, this would elicit an approximately 2.62 pA/pF current in males and approximately 1.51 pA/pF current in female β cells. Because Ca^2+^ influx at –60 mV was approximately –1 pA/pF in β cells of both sexes, the larger K_v_2.1 current would maintain the male β cell MP close to the K^+^ reversal potential over a wider range of extracellular glucose concentrations.

This change in MP will also modulate the incretin insulinotropic effects. Recently, it has been shown that persistent β cell membrane depolarization shifts the incretin pathway from G_s_ to G_q_ (57). Although female β cells are not continuously depolarized but show only a stronger glucose-dependent MP depolarization, this is sufficient to increase the contribution of the G_q_ pathway to the GLP-1–induced potentiation while maintaining a similar total incretin response. An additional effect of a partial K_v_2.1 channel inhibition has previously been demonstrated to be the reduced GLP-1 dose required to trigger an incretin insulinotropic effect, both in isolated healthy islets as well as a diabetes mouse model (42). Therefore, it is very tempting to speculate that due to the more depolarized MP, females might respond with a similar insulinotropic effect compared to males but using a lower dose that will minimize the side effects. Our data also suggest that gastric inhibitory peptide (GIP) will have a reduced effect in females since GIP activates only G_s_, while GLP-1 activates both G_s_ and G_q_ pathways (57).

Besides their role in membrane hyperpolarization, K_v_2.1 channels can modulate β cell function and survival through other mechanisms. It has been shown that K_v_2.1 channels increase vesicle exocytosis via enhanced vesicle recruitment and fusion (44–46). However, our data do not show a sex-specific role of K_v_2.1 channel in exocytosis since Cm measurements as well as first-phase insulin release were similar in β cells of both sexes. Nevertheless, a structural and sex-specific role of K_v_2.1 channels has been demonstrated in smooth muscle cells (73). In male myocytes, the K_v_2.1 channels are expressed at lower levels and mostly involved in membrane hyperpolarization, while in female myocytes K_v_2.1 channels show higher expression levels but act as an enhancer of Ca_V_1.2 L-type Ca^2+^ channel clustering and therefore increase Ca^2+^ influx and myogenic tone. Whether K_v_2.1 channels assume a similar sex-specific structural role in pancreatic β cells remains to be investigated. We also cannot distinguish whether the sex-specific K_v_2.1 current density is caused by sex hormone–modulated transcriptional regulation, subcellular localization, protein interactions, or posttranslational modifications. Nevertheless, we can exclude an acute effect of sex hormones on K_v_2.1 gating properties since all our experiments were performed on pancreatic islets and β cells cultured for 24 hours in the absence of any sex hormones.

In addition to the different K_v_2.1 conductances, the Ca^2+^-activated BK and SK currents also showed sex-specific properties. In response to a short (90 ms) Ca^2+^-loading prepulse, the SK component showed a significantly higher amplitude in females compared with males. Functionally, the larger SK currents explain the higher amplitude of the AHP component observed in single APs recorded during the induced electrical activity in response to step current injections. The BK currents had the same amplitude in β cells of both sexes, but showed a significantly faster inactivation in females compared with males following Ca^2+^-loading prepulses shorter than 810 ms. The faster BK current inactivation could partially be responsible for the increased bursting behavior and burst duration observed in female β cells. However, both SK and BK currents following longer Ca^2+^-loading prepulses showed the same biophysical properties in β cells of both sexes, suggesting that in pancreatic β cells the sex hormone regulation leads to a different functional coupling between the Ca^2+^ source and the SK or BK K^+^ channels.

A caveat of our study is that we performed our analysis on mice 5 to 19 weeks old. Recently it has been shown that sex-specific pancreatic β cell mass and glucose tolerance are age dependent (74). Male mice showed glucose and insulin intolerance comparable to those of females at 3 and 6 months of age, while 6-month-old males displayed increased β cell mass in response to reduced insulin resistance compared with littermate females (74). However, recently a different study reported that until 3 months both male and female C57BL/6 mice have similar insulin sensitivity (60). Additionally, although the 14-week-old male mice used in our study did show a tendency toward larger islet areas compared with females (male islet size = 0.9 ± 0.11 μm^2^, *n* = 89; female islet size =0.55 ± 0.02 μm^2^, *n* = 566), this difference was not significantly different (Mann-Whitney test *P* = 0.14). We did not quantify the total β cell mass by multiplying the relative insulin-positive area with pancreas weight. But, important for our study is that the islet area and β cell contribution to islets did not show any significant sex-specific differences and therefore we can assume a similar islet cell paracrine modulation (75–78).

A second limitation of our study is that we performed our analysis only on 1 mouse strain, C57BL/6 × 129J. Several previous studies have demonstrated significant strain-specific differences in mice regarding insulin release (79, 80), pancreas size and structure (81), and susceptibility to diabetes (79–84). However, our findings showing that female islets secrete more insulin that leads to a better glucose tolerance corroborate well both with human data (2, 60–63) and several rodent studies (60, 74, 85). Additionally, a recent study demonstrated (74), also in the C57BL/6 background, that female islets respond with reduced Ca^2+^ transients to glucose stimulation but increased β cell oscillatory responses compared with males, very similar to our previous (48) and current study. Nevertheless, while under basal glucose conditions female islets secreted more insulin, the stimulated insulin release was higher in males (74). The higher insulin release in males could be secondary to already existing altered insulin sensitivity and not directly caused by sex-specific β cell functions. Given the different mouse genetic backgrounds and different experimental conditions between laboratories, it is not surprising that the results are sometimes divergent. However, one constant is that sex plays a critical and very complex role in glucose metabolism, affecting insulin sensitivity, β cell mass and its resilience to diabetes, and insulin synthesis and release.

Our study demonstrates dramatic sex-specific differences in β cell MPs and electrical activity. These differences are primarily caused by significantly different sex-specific K_v_2.1 current densities. In male mice, the larger K_v_2.1 currents cause a reduced β cell firing frequency that translates to reduced second-phase insulin release and therefore increased susceptibility to diabetes. In female β cells, the smaller K_v_2.1 current density causes a stronger glucose-induced membrane depolarization that increases the firing frequency but limits the Ca^2+^ influx due to MP-dependent Ca_V_ channel inactivation. Under stress conditions, the smaller-amplitude Ca^2+^ transients might limit mitochondrial damage and therefore, increased female β cell resilience. Additional studies are required to investigate how the differences that we observe here might contribute to sex-specific incidence of hyperglycemia in older ages, especially after the onset of insulin resistance.

## Methods

### Sex as a biological variable.

We conducted this study on pancreatic islets and β cells of both male and female mice.

### Mouse model.

The mouse line has a mixed C57BL/6 × 129J background (48). The mouse colony maintenance and all experiments were performed in conformity with international laws and respected the 3R principle. The data presented in this manuscript did not include any animal experimentation. The number of mice used for tissues collection was regularly reported to the Austrian Ministry of Science (BMWFW).

### Islet and β cell isolation.

Pancreatic islets from adult mice (5–19 weeks old) were enzymatically isolated as previously reported (48, 86). For electrophysiology, insulin release, and Ca^2+^ imaging recordings the islets were cultured at 37°C and 5% CO_2_ overnight in RPMI 1640 medium (11 mM glucose) supplemented with 5% FBS, 2 mM L-glutamine, 100 mg/mL streptomycin, and 100 IU/mL penicillin. For the experiments performed on isolated β cells, the islets were incubated in solution containing (in mM) 138 NaCl, 6 KCl, 3 MgCl_2_, 5 HEPES, 3 glucose, 1 EGTA, and 1 mg/mL BSA for 12 minutes at 37°C and then mechanically dissociated and cultured as previously described (48, 86).

### Solutions for electrophysiology.

For membrane potential recordings as well as K_V_ and K_ATP_ K^+^ currents a physiological solution containing (in mM) 140 NaCl, 3.6 KCl, 2 NaHCO_3_, 0.5 NaH_2_PO_4_, 0.5 MgSO_4_, 5 HEPES, 2.5 CaCl_2_, glucose (2, 5, 7.5, 10, 15, or 20) (pH 7.4 with NaOH) was used. The internal solution consisted of (in mM) 76 K_2_SO_4_, 10 NaCl, 10 KCl, 1 MgCl_2_, and 5 HEPES (pH 7.35 with KOH). For K_2_P currents, the extracellular solution contained in (in mM) 97.7 *N*-methyl-D-glucamine, 26 KCl, 25 HEPES, 1.2 MgSO_4_, 1.2 KH_2_PO_4_, 14.4 glucose, 20 tetraethylammonium chloride (TEA-Cl), and 0.1 tolbutamide (Sigma-Aldrich) (pH 7.35 with NaOH). The intracellular solution was composed of (in mM) 140 KCl, 1 MgCl_2_, 10 EGTA, and 10 HEPES (pH 7.25 with KOH). For Ca_V_ currents, the pipette was filled with (in mM) 76 CsSO_4_, 10 CsCl, 10 KCl, 1 MgCl_2_, and 5 mM HEPES. For the capacitance measurements, the pipette solution contained (in mM) 140 CsCl, 10 NaCl, 1 MgCl_2_, 0.05 EGTA, 5 HEPES, 0.1 cAMP, and 0.1 MgATP (pH 7.2 with CsOH). The external solution for Ca_V_ currents and capacitance measurements contained (in mM) 118 NaCl, 5.6 KCl, 20 TEA-Cl, 1.2 MgCl_2_, 5 HEPES, 2.6 CaCl_2_, and 5 glucose (pH 7.4 with NaOH).

### Glucose-induced and current injection–induced electrical activity.

The electrical activity was measured in current-clamp mode using the HEKA amplifier and the perforated patch-clamp technique (0.24 mg/mL amphotericin B, Sigma-Aldrich) in intact islets maintained at 32°C–34°C by perfusing increasing glucose concentrations (2, 5, 7.5, 10, 15, and 20 mM). β Cells were identified by being active at glucose concentrations above 5 mM and silent at lower concentrations and/or by the steady-state inactivation of Na^+^ currents, as previously described (21). To characterize the current-step-induced electrical activity, islets were dispersed to obtain isolated single cells, as previously described (86).

### Voltage-clamp experiments.

All voltage-clamp experiments were performed at room temperature. The Na_V_, BK, SK, K_ATP_ K_2_P, and K_V_2.1 currents were recorded in β cells in intact pancreatic islets. The Ca_V_ currents were recorded in isolated β cells. The Na_V_, Ca_V_, BK, SK, and K_V_2.1 current were subject to online P/4 leak subtraction, while the K_ATP_ and K_2_P were not leak subtracted. For this analysis, only recordings with a leak of less than –50 pA were used. The current densities were calculated by dividing the measured current by the Cm that represents a direct measurement of cell area. The Cm did not show a sex-specific difference in all our experiments (Cm_Males_ = 6.74 ± 0.18, *n* = 78; Cm_Females_ = 7.42 ± 0.23 pF, *n* = 88; *P* = 0.09 by Mann-Whitney test). The voltage dependence of the Na^+^ current inactivation was used to distinguish the β cells from pancreatic non-β cells (48). K_ATP_ currents were recorded using a double square pulse of ± 10 mV (500 ms long) starting from –70 mV. Diazoxide (340 μM, Sigma-Aldrich) was dissolved in 0.1 mM NaOH solution and was added to the bath solution. For recording 2-pore K^+^ channels (K_2_P), a 1-second-ramp protocol from –120 mV to +60 mV was applied. The Ca^2+^-activated K^+^ channels and K_v_ isoforms were pharmacologically dissected using the specific BK (paxilline, 1 μM; Sigma-Aldrich) and SK (apamin, 200 nM; Alomone) blockers. The protocol used for recording Ca^2+^-activated K^+^ channels consists of a Ca^2+^-loading prepulse to 0 mV that increased its duration by 90 ms every step. The test pulse consisted of a depolarization to +80 mV for 600 ms. To determinate the K_v_ isoform, we applied the specific K_v_2.1 blocker Strx-1 (100 nM; Smartox Biotechnology). Ca^2+^ currents were elicited using 200-ms depolarization steps from –80 mV or –50 mV to +60 mV in 10-mV increments and analyzed as previously described (48).

### Capacitance measurements.

The increase in Cm was recorded in β cells in intact islets bathed at 32°C–33°C using the lock-in and sine+DC mode of the HEKA amplifier. The exocytosis was triggered by a train of 10 depolarizing steps, 500 ms long, from –70 mV to 0 mV.

### Ca^2+^ imaging.

Intact pancreatic islets were loaded with Fluo-4-AM Ca^2+^ indicator (5 μM; Thermo Fisher Scientific) and 0.1% pluronic acid for 30 minutes at room temperature followed by a 30-minute incubation at 37°C. Single islets were perfused with physiological solution in which Ca^2+^ was 2.5 mM or omitted. Carbachol (0.2 mM; Sigma-Aldrich) was added to deplete the ER by activating IP_3_Rs in 0 mM Ca^2+^–containing extracellular solution.

### Dynamic insulin release.

For each experiment, 20 islets were perfused with a physiological solution (in mM) of 140 NaCl, 3.6 KCl, 2 NaHCO_3_, 0.5 NaH_2_PO_4_, 0.5 MgSO_4_, 5 HEPES, and 2.5 CaCl_2_ with different glucose concentrations (2, 5, and 10 mM) at 37°C. The flow rate was set to 200 μL/min and the fractions collected every 2 minutes using an automated system. Insulin concentration in the fractions was measured with an ultrasensitive sandwich ELISA protocol. GLP-1 (1 nM; Bachem) and YM-254890 (100 nM; MedChemExpress) were added where indicated. To allow a direct comparison between different experiments, the insulin release per islet and unit of time was calculated based on islet area (87) and flow rate respectively. A picture of all the islets was obtained before the experiment using the Moticam 2500 camera connected to a fixed-focus dissection microscope. The area was calculated using the proprietary Moticam software that incorporates a calibrated distance measurement. The insulin concentration was multiplied by the correction factor representing the relative difference in islet area compared with the area of the largest islet group measured in all experiments.

### Statistics.

Data analysis was performed using FitMaster (Heka), Clampfit 10.7 (Axon Instruments), SigmaPlot 13 (Systat Software, Inc.), Origin (2021b), or GraphPad Prism (8.01). All values are presented as mean ± SEM for the indicated number of cells (*n*), except where stated otherwise. For all experiments, a minimum of 3 mice per sex were used. Statistical significance was calculated using paired or unpaired Student’s *t* test, Mann-Whitney test, or 1-way ANOVA followed by Bonferroni’s post hoc test.

### Data and materials availability.

All analyzed data are available in the main text. The raw data can be found in the Supporting Data Values file in the supplemental material.

## Author contributions

NJP and PT conceptualized the study. NJP, TT, SMG, and PT developed the methodology. NJP and TT carried out experiments. NJP and PT generated figures. PT supervised the study. NJP wrote the original draft of the manuscript, which was reviewed and edited by NJP and PT.

## Supplementary Material

Supplemental data

Supporting data values

## Version 1 02/15/2024

In-Press Preview

## Version 2 03/22/2024

Electronic publication
