# Supplementary material for: Molecular mechanism responsible for sex differences in electrical activity of mouse pancreatic β cells
Source: JCI Insight. 2024 Feb 15;9(6):e171609. doi: 10.1172/jci.insight.171609 (PMC11063940; doi:10.1172/jci.insight.171609)
Supplement: Supplemental data [file jciinsight-9-171609-s233.pdf]

Molecular mechanism responsible for sex differences in electrical activity of mouse pancreatic β-cells

Noelia Jacobo-Piqueras[1], Tamara Theiner[1], Stefanie M. Geisler[1], Petronel Tuluc[1*]

Supplementary Figures

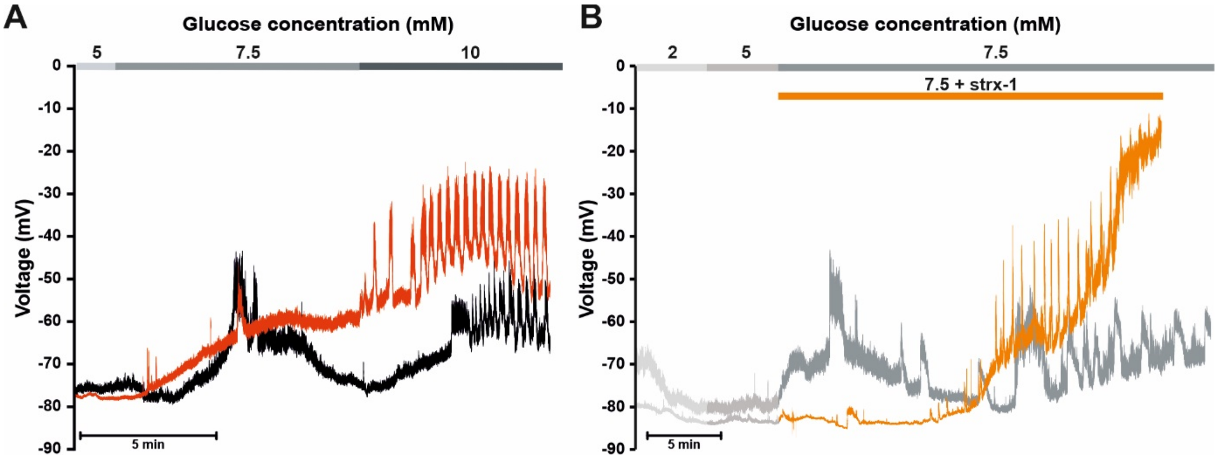

Figure S1. Sample traces of glucose-induced electrical activity. A. Increasing extracellular glucose concentartion induces a steady membrane depolarization in female (red) but not male (black) β-cells. Note that male β-cell MP remains at a hyperpolarized value even in the presence of 10 mM extracellular glucose. **B.** Sample traces of the glucose induced electrical activity in male β-cells from intact islets stimulated with 2, 5 and 7.5 mM glucose without stromatoxin-1 (grey) and in presence of stromatoxin-1 (orange).

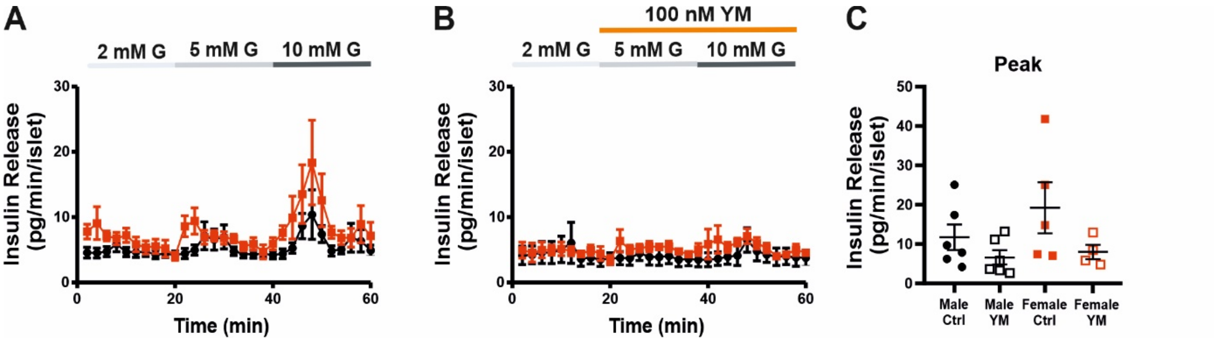

Figure S2. Inhibition of Gq pathway alone equally reduces glucose-induced insulin secretion in male and female islets. (A) Dynamic insulin release from male (n=6) and female (n=5) islets (20 islets/experiment) in 2, 5 and 10 mM glucose. **(B)** Dynamic insulin release from male (n=6) and female (n=4) islets in 2, 5 and 10 mM glucose plus 100 nM of the Gq inhibitor, YM-254890. **C.** Maximum insulin release values for the expeiments presented in panels A and B (Peak male: Ctrl: 11.73 ± 3.24, YM: 6.61 ± 1.823 P=0.1994; Peak female: Ctrl: 19.22 ± 6.51, YM: 8.01 ± 1.81, P= 0.19). All values are means ± SEM. Statistics with Mann-Whitney test.
